# Interleaved ^31^P MRS/^1^H ASL for analysis of metabolic and functional heterogeneity along human lower leg muscles at 7T

**DOI:** 10.1002/mrm.28088

**Published:** 2019-12-17

**Authors:** Fabian Niess, Albrecht Ingo Schmid, Wolfgang Bogner, Michael Wolzt, Pierre Carlier, Siegfried Trattnig, Ewald Moser, Martin Meyerspeer

**Affiliations:** ^1^ Center for Medical Physics and Biomedical Engineering Medical University of Vienna Vienna Austria; ^2^ High Field MR Center Medical University of Vienna Vienna Austria; ^3^ Department of Biomedical Imaging and Image‐guided Therapy Medical University of Vienna Vienna Austria; ^4^ Department of Clinical Pharmacology Medical University of Vienna Vienna Austria; ^5^ NMR Laboratory Institute of Myology Paris France

**Keywords:** ^31^P MRS, energy metabolism, interleaved acquisition, muscle exercise, perfusion, pH

## Abstract

**Purpose:**

MR offers the unique possibility to noninvasively investigate cellular energy metabolism via 31P MRS, while blood perfusion, which provides oxygen and substrates to the tissue, is accessible by arterial spin labeling (ASL) 1H MRI. Because metabolic and hemodynamic parameters are linked, it would be desirable to study them simultaneously. A 3D‐resolved method is presented that allows such measurements with high spatiotemporal resolution and has the potential to discern differences along an exercising muscle.

**Methods:**

Multi‐voxel localized ^31^P MRS was temporally interleaved with multi‐slice pASL 1H MRI. Phosphorus spectra were collected from two adjacent positions in gastrocnemius medialis (GM) during rest, submaximal plantar flexion exercise and recovery, while perfusion and $T_{2}^{*}$‐weighted axial images were acquired at the same time. Seventeen healthy volunteers (9 f / 8 m) were studied at 7 T.

**Results:**

An increase of postexercise perfusion and $T_{2}^{*}$‐weighted signal in GM positively correlated with end‐exercise PCr depletion and pH drop. At proximal positions functional and metabolic activity was higher than distally, that is, perfusion increase and peak $T_{2}^{*}$‐weighted signal, end‐exercise PCr depletion, end‐exercise pH, and PCr recovery time constant were significantly different. An NOE‐induced SNR increase of approximately 20 % (*P* < .001), at rest, was found in interleaved ^31^P spectra, when comparing to ^31^P‐only acquisitions.

**Conclusions:**

A technique for fast, simultaneous imaging of muscle functional heterogeneity in ASL, $T_{2}^{*}$ and acquisition of time‐resolved ^31^P  MRS data is presented. These single exercise recovery experiments can be used to investigate local variations during disease progression in patients suffering from vascular or muscular diseases.

## 1 INTRODUCTION

In exercising calf, the workload is distributed between individual muscles according to their biomechanical and anatomical properties.^1^ This leads to heterogeneous activation patterns across the limb with spatially distinct metabolic activity and perfusion. The extent of the metabolic activation in one specific muscle is determined by many factors, for example, prevalence of muscle fiber types, exercise intensity, individual training state, and oxygen supply.^2^, ^3^ Sufficient blood perfusion for substrate and oxygen transport to the tissue is crucial for maintaining cell function and tissue health and has been linked to the muscles’ energy metabolism.^4^ Additionally, various vascular and metabolic diseases, such as diabetes mellitus^5^, ^6^ and peripheral arterial disease,^7^ impair both tissue perfusion and the energy metabolism in mitochondria of skeletal muscles.^8^, ^9^

These processes have been investigated in several studies employing either ^31^P MRS and MRI^10^, ^11^, ^12^, ^13^, ^14^ or ^1^H arterial spin labeling (ASL) MRI^15^, ^16^, ^17^, ^18^ in calf muscles during and after exercise. ^1^H ASL is an approach to noninvasively assess local perfusion rates,^19^, ^20^ while ^31^P MRS is a well‐established method to investigate the energy metabolism^21^ in vivo. Physiologically relevant parameters can be derived from time courses of phosphocreatine and inorganic phosphate, that is, pH, PCr recovery time constant $\tau_{\mathrm{PCr}}$ and maximum oxidative capacity $Q_{\max}$,^22^, ^23^ which are linked to muscle activation and can give insight into intracellular oxidative ATP production.^24^ To improve the understanding of physiology in human skeletal muscle tissue with the help of MR, a meaningful model of the interplay of the accesible parameters has to be derived. To achieve this, the dynamics of functional and metabolic processes within a specific muscle volume need to be investigated accurately, with sufficient quality and, at best, simultaneously. The MR acquisition scheme hence has to deliver multi‐nuclear data with high temporal resolution and spatial selectivity, while delivering sufficient SNR.

Simultaneous acquisition of ^1^H and ^31^P data during a single time‐resolved experiment involving muscle exercise and recovery is feasible without mutual saturation, as shown by Wray et al^4^ who studied muscle perfusion and metabolism during exercise in young and elderly subjects by combining single‐slice ^1^H ASL with unlocalized ^31^P MRS at 4 T.

In most studies published so far, the recruitment of tissue along a specific muscle was implicitly assumed to be homogeneous, largely ignoring potential spatial inhomogeneities within, and often even between adjacent muscles. Using ^31^P MRS and ^1^H MRI in separate experiments Boss et al^25^ recently discovered variations of oxidative capacity not only between muscle groups, but also along the studied muscle, (tibialis anterior). This heterogeneity was confirmed in a follow‐up study, reporting a gradient of perfusion and $O_{2}$ supply measured with NIRS, intra‐voxel incoherent motion imaging (IVIM) and ^31^P MRS in consecutive experiments.^26^

Here, we present a novel pulse sequence that combines multi‐slice ^1^H ASL MRI with multi‐voxel localized ^31^P MRS in a single, time‐resolved measurement. The method was developed for application at ultra‐high field, with the goal of investigating the combined functional and metabolic exercise response of healthy human muscle and studying its heterogeneity along the calf.

## 2 METHODS

^31^P MR spectra were acquired from two positions along the gastrocnemius medialis (GM) of healthy volunteers during rest, plantar flexion exercise, and recovery, that is, two volumes of interest (VOI) were placed adjacently to each other at a more proximal and a more distal position in GM. Simultaneously with ^31^P MR spectra, a series of axial perfusion and $T_{2}^{*}$‐weighted images were acquired from the calf, using multi‐slice ^1^H ASL, covering both ^31^P volumes.

### 2.1 Volunteers

Seventeen healthy subjects (body mass index = 22 ± 2 kg/$m^{2}$; age = 25 ± 4 years; 9 f / 8 m) volunteered to take part in the study and declared written informed consent to the protocol, which is in accordance with the guidelines of the local ethics committee and the latest version of the declaration of Helsinki. All subjects were recreationally physically active.

### 2.2 Experimental setup

The measurement protocol consisted of two parts: preparation time of about 25 minutes followed by the dynamic exercise recovery protocol of another 15 minutes. The preparation included placement of the subject in the scanner, measurement of maximum voluntary contraction force (MVC), placement of ^1^H imaging slices and ^31^P spectroscopy voxels and the acquisition of ^1^H/^31^P reference scans (needed for normalization of ASL images and calculation of ^31^P SNR without ^1^H interleaving). The dynamic exercise recovery protocol was 2 minutes rest, 3 minutes plantar flexion exercise and 10 minutes recovery time. Data were continuously acquired throughout the dynamic experiment. A nonmagnetic pneumatic ergometer,^27^ exclusively designed for MR measurements, was used. The pneumatic system was inflated using a manual pump to adjust the exercise intensity to 30 % of the subjects’ MVC, which was measured on the same ergometer prior to the experiment. Subjects were instructed to time the plantar flexion exercise within the 3 s time window between the semi‐LASER acquisitions, which was sufficiently long to perform two pedal pushes.

A double‐tuned surface coil transceiver array with two ^1^H channels (*d* = 17 cm, *l* = 12.5 cm) and three ^31^P channels (*d* = 15 cm, *l* = 10 cm), shaped to the anatomy of the human calf,^28^ was used in a 7 T Magnetom whole‐body MR system (Siemens, Erlangen, Germany). The receiver frequency was switched for reception of ^1^H and ^31^P Larmor frequencies using a custom‐built interleaving device.^29^

### 2.3 ^1^H ASL MRI

Symmetric pulsed arterial spin labeling (PASL) based on flow‐sensitive alternating inversion recovery (FAIR)^18^ was applied, that is alternating global (tag) and slice‐selective (control) inversion of the readout slab using an adiabatic BASSI pulse (bandwidth‐modulated adiabatic RF pulse for uniform selective saturation and inversion). This type of inversion pulse features a good tagging efficiency and stability at 7 T, as shown by Schewzow et al.^18^, ^30^ The blood inflow time after inversion was 1.3 s followed by a 200 ms Q2TIPS saturation scheme^31^ to prevent blood inflow into the imaging planes shortly before readout. Ten echo‐planar imaging slices ($T_{E}\;=\;20\;\text{ms}$, *d* = 6 mm) were acquired sequentially in descending order (direction: head‐foot) without slice gaps, with $T_{R}$ = 6 s. Perfusion and $T_{2}^{*}$‐weighted images were calculated by either subtraction or pair‐wise averaging of control and tag images, resulting in an image time series with a resolution of 12 s. Due to the sequential multi‐slice EPI readout the inversion time accumulated additional 50 ms for every consecutive slice. Therefore, perfusion values were corrected per slice for this delay using a model based on Calamante et al.^32^ Regions of interest (ROI) were defined for three muscles (gastrocnemius medialis, gastrocnemius lateralis, and soleus) in all 10 slices. The average of the perfusion signal and $T_{2}^{*}$‐weighted image amplitude was calculated for each ROI and time point, and a moving average filter covering three time points was applied for calculating the respective time courses for each muscle. To avoid overestimation of possible outliers, postexercise perfusion was defined as mean ± SD over 4 minutes during recovery. The time window of averaging was chosen as 60 ‐ 300 s postexercise to compensate for possible motion artifacts during exercise. Since $T_{2}^{*}$‐weighted time courses are hardly ever affected by outliers, postexercise peak values were chosen for comparison between muscles.

To investigate potential systematic errors of the multi‐slice protocol, perfusion and $T_{2}^{*}$‐weighted data derived from the multi‐slice acquisition scheme were compared to data that were acquired with a single‐slice ^1^H ASL protocol. For this comparison, four subjects were reinvited on a different day to acquire axial images using a single‐slice ASL acquisition scheme from two positions consecutively, in separate exercise recovery measurements (proximo‐distal distance: 3.5 cm, corresponding to the space between the ^31^P voxels). The coil was always positioned in the magnet’s iso‐center and the imaging, inversion and saturation slices were identical in the first and second acquisition, while the subject’s leg, ergometer, and patient bed were translated to ensure that the only difference between measurements was the muscle position relative to the imaging volume and coil. That way, the entire MR sequence protocol was repeated, with identical tagging volumes, transmit and receive $B_{1}$ field and the same position relative to the magnet and gradient system. The proximal position was always measured first, and the time between the exercises for the repeated experiment was at least 33 minutes.

The space between distal and proximal single‐slice positions (3.5 cm) corresponded to a distance of six axial slices in the multi‐slice protocol (slice thickness of 6 mm). Accordingly, results of distal and proximal single‐slice acquisitions were compared to results of slices 4 and 10 acquired with the multi‐slice protocol.

### 2.4 ^31^P MRS

**Figure 1:**
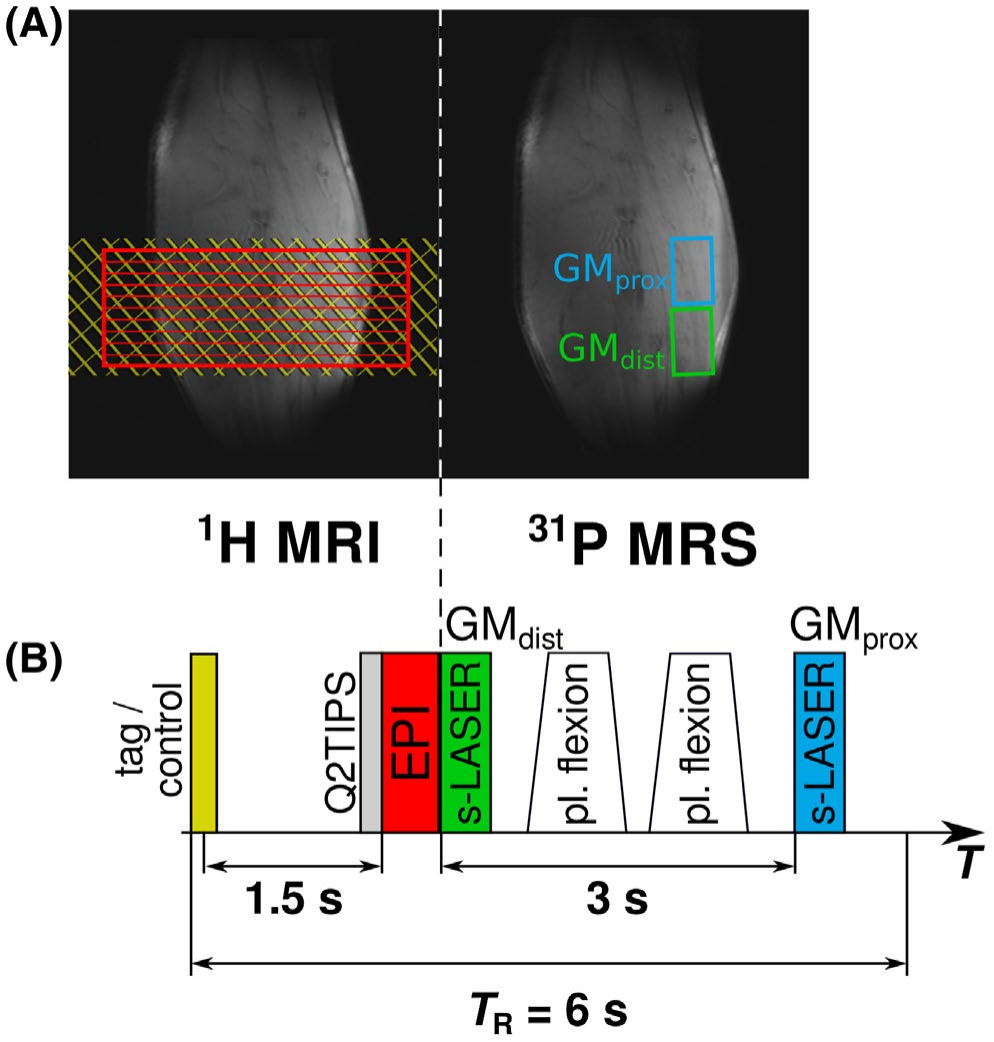
Coronal gradient‐echo images of the calf and typical placement of the adiabatic inversion slab (yellow), ten ^1^H imaging slices (red) and ^31^P VOIs at distal (green) and proximal (blue) position in gastrocnemius medialis (A). Schematic diagram of the pulse sequence including ^1^H MRI and ^31^P MRS, acquiring a full set of images and spectra every $T_{R}\;=\;6\;s$ (B). ^1^H MRI comprises arterial spin labeling using FAIR tagging (alternating slice selective and global inversion) and Q2TIPS saturation schemes with multi‐slice EPI readout. ^31^P MR spectra are acquired from GM at two positions using multi‐voxel semi‐LASER ^31^P MRS

To acquire ^31^P MR spectra, a multi‐voxel semi‐LASER acquisition scheme was applied using similar parameters as recently published^33^ (pulse durations: $d_{\mathrm{exc}}\;=\;2.6\;\text{ms}$, $d_{\mathrm{refoc}}\;=\;4.5\;\text{ms}$, echo time: $T_{E}\;=\;29\;\text{ms}$, flip angle in the region of interest *α* ≈ 90°, receiver bandwidth = 5000 Hz, vector size = 2048 complex points after removing oversampling). Spectra from two independent volumes placed in GM (${\mathrm{VOI}}_{\mathrm{distal}}\;=\;27\;\pm \;6\;\text{mL}$, ${\mathrm{VOI}}_{\mathrm{proximal}}\;=\;32\;\pm \;7\;\mathrm{mL}$) were acquired directly after the ^1^H image readout with 3 s delay between the acquisitions (cf. Figure 1). To minimize signal loss with the multi‐voxel acquisition, the excitation and refocusing slices were oriented such that a pair of refocussing slices overlapped fully, and overlap of excitation slices between voxels was avoided enitrely.^33^

The evolution of phosphocreatine (PCr) and inorganic phosphate (Pi) resonances were quantified during rest, plantar flexion exercise, and recovery. PCr depletions were calculated by normalizing PCr values to the median of the last minute of recovery for every data set. End‐exercise PCr depletion ($d_{\mathrm{PCr}}$) was defined as mean ± SD of the last 30 s of plantar flexion. A mono‐exponential curve was fitted to PCr data during recovery to calculate the PCr recovery time $\tau_{\mathrm{PCr}}$. The Henderson‐Hasselbalch equation using the chemical shift between PCr and Pi was applied to calculate the pH from each pair of consecutive acquisitions averaged.

### 2.5 Nuclear overhauser effect

Figure 1 shows a sequence diagram of each repetition ($T_{R}\;=\;6\;s$) consisting of the ^1^H ASL part, which requires 2 s, followed by a multi‐voxel ^31^P semi‐LASER acquisition scheme^33^ within the remaining 4 s. This allows for acquiring ^1^H data of the calf together with ^31^P spectra of the GM at the distal and proximal half of the ^1^H inversion slab. The full overlap of slice selective ^1^H inversion and ^31^P VOIs during the control acquisition of ASL enhances the signal‐to‐noise ratio (SNR) of ^31^P spectra due to the ^1^H/^31^P nuclear Overhauser effect (NOE) and avoids SNR alterations compared to global inversion during the tag acquisition. The SNR enhancement was investigated by acquiring resting ^31^P spectra without ^1^H interleaving.

### 2.6 Postprocessing

Image reconstruction of ^1^H data were performed using the MR scanner’s native “Image Calculation Environment” (ICE) followed by a postprocessing pipeline in MATLAB (Mathworks, Natick, Massachusetts) for calculation of perfusion and $T_{2}^{*}$‐weighted multi‐slice images and time courses. ^31^P MR spectroscopy data were extracted and processed from raw data using in‐house developed Python scripts (http://www.python.org) for phasing and channel combination. Signals were phased to the highest peak magnitude of PCr in the frequency domain after 7 Hz Lorentzian apodization and 2× zero‐filling. The channel combination was then performed by weighted averaging of the raw data (that is, without apodization and zero‐filling). Weights were calculated as proportional to signal, averaged over four resting spectra (excluding the fully relaxed spectrum).

Phosphocreatine and inorganic phosphate resonances were quantified and pH time courses were calculated using jMRUI^34^ with the fitting routine AMARES.^35^

Since the signal from the distal voxel in GM was always acquired approximately 3 s after plantar flexion (see Figure 1), end‐exercise PCr depletion values were corrected retrospectively for initial recovery during these 3 s using the individually derived recovery time constant $\tau_{\mathrm{PCr}}$.

SNR was calculated using the partially saturated resting spectra of each time series by dividing the PCr peak amplitude by the standard deviation of the signal in a region containing only noise, 15 ppm off‐center across 1/16 of the total bandwidth. Linewidths were taken from the AMARES fit of the PCr peak.

For correlating ^1^H MRI with ^31^P MRS, data from ROIs in imaging slices that overlapped with a spectroscopy voxel were averaged per location, that is, slices 3 – 6 were compared to the distal and slices 7 – 10 to the proximal ^31^P voxel by calculating linear regressions between derived parameters accordingly.

### 2.7 Statistical tests

Statistical tests were performed using Python (http://www.python.org) to test for differences between positions. Wilcoxon signed‐rank test was applied on ^31^P results between distal and proximal voxel and Friedman test for repeated measurements on ^1^H results between all relevant imaging slices. For multiple comparisons Nemenyis post hoc tests were used. Bland‐Altman plots were created and the Pearson product‐moment correlation was calculated, as a measure for inter‐method (single‐slice vs multi‐slice) and test‐retest reliability according to Murphy et al.^36^ For the latter, two subjects were remeasured, to test the repeatability of the interleaved ^1^H/^31^P acquisition scheme.

## 3 RESULTS

The force applied on the pedal during exercise was on average 196 ± 37 N with a corresponding power output of 5 ± 1 W.

### 3.1 ^31^P MRS

**Figure 2:**
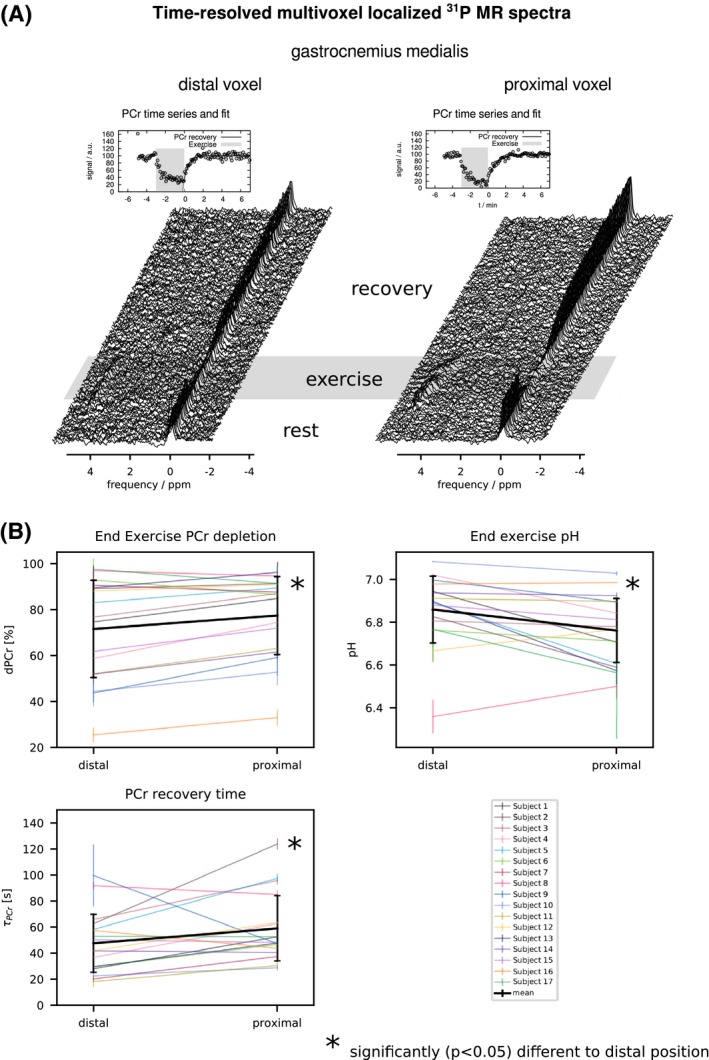
Time series of ^31^P spectra simultaneously acquired during rest, plantar flexion exercise and recovery in a distal and proximal VOI in gastrocnemius medialis. The inserts show the corresponding mono‐exponential PCr recovery fits (A). End‐exercise PCr depletion ($d_{\mathrm{PCr}}$), end‐exercise pH, and PCr recovery time constants ($\tau_{\mathrm{PCr}}$) for each subject at distal and proximal position (B). The bold line represents mean ± SD

We successfully acquired data on the evolution of PCr and inorganic phosphate with a time resolution of 6 s during rest, plantar flexion exercise and recovery (see Figure 2A). Time courses of PCr and pH were used to derive metabolically relevant parameters, that is, PCr depletion and pH at the end of exercise, and the recovery time constant of PCr ($\tau_{\mathrm{PCr}}$). Averaged and individual results from two positions in GM over seventeen subjects are shown in Figure 2B and Table 1.

**Table 1:** Parameters derived from data acquired at distal and proximal position in GM using the interleaved ^1^H/^31^P acquisition scheme: voxel size, linewidth, end‐exercise PCr depletion ($d_{\mathrm{PCr}}$), end‐exercise pH (${\mathrm{pH}}_{\mathrm{ee}}$), calculated PCr recovery time constant ($\tau_{\mathrm{PCr}}$), postexercise perfusion (averaged 60 ‐ 300 s), and $T_{2}^{*}$‐weighted peak values given as mean ± SD over all subjects

^31^P MRS	${\mathrm{GM}}_{\mathrm{distal}}$	${\mathrm{GM}}_{\mathrm{proximal}}$
Voxel size (mL)	27 ± 3	32 ± 7
Linewidth (Hz)	10 ± 3	12 ± 4^a^
End‐exercise PCr depletion (%)	71 ± 21	77 ± 18^a^
${\mathrm{pH}}_{\mathrm{ee}}$	6.86 ± 0.16	6.76 ± 0.15^a^
$\tau_{\mathrm{PCr}}$ (s)	48 ± 23	59 ± 26^a^
**^1^H MRI**	$G{M_{\mathrm{slice}}}_{3}$	$GM_{\mathrm{slice}10}$
Perfusion signal (a.u.)	23 ± 13	46 ± 24^b^
$T_{2}^{*}$‐weighted peak (a.u.)	1.05 ± 0.06	1.09 ± 0.06^b^

a Significantly different to ${\mathrm{GM}}_{\mathrm{distal}}$.

b Significantly different to ${\mathrm{GM}}_{\mathrm{slice}3}$.

Significant differences between proximal and distal positions in GM were found for derived ^31^P parameters, that is, in the proximal VOI the end‐exercise depletion was higher (*P* < .005), end‐exercise pH was lower (*P* < .01) and PCr recovery time $\tau_{\mathrm{PCr}}$ was longer (*P* < .05) than at the distal location. Differences in PCr recovery times are presumably caused by the known influence of pH on $\tau_{\mathrm{PCr}}$.^37^

SNR in interleaved ^31^P data was significantly higher, with 38 ± 13 (*P* < .001) at rest, than in ^31^P spectra acquired in additional scans without interleaving (SNR = 32 ± 13). The linewidths, however, were not significantly different (*P* = .145).

Averaged ^31^P MRS results from subject no. 6 and 7 of the repeatability test are presented in Supporting Information Table S1.

### 3.2 ^1^H MRI

**Figure 3:**
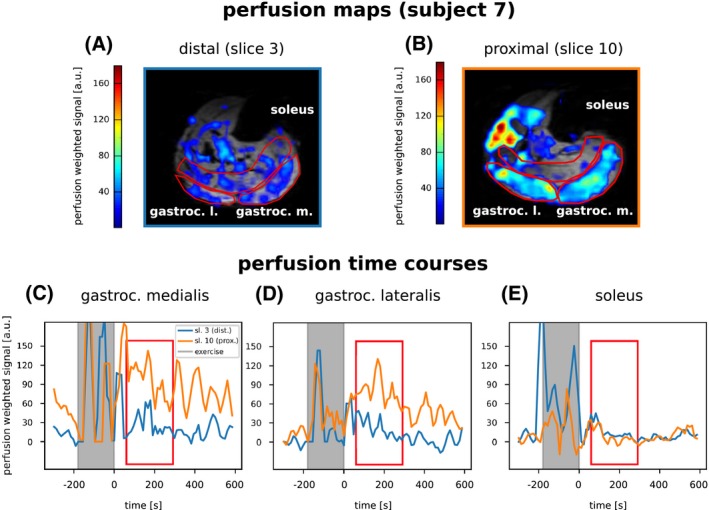
Perfusion maps at distal (A) and proximal (B) position overlaid with transversal EPI slices of the calf. Corresponding perfusion time courses of three ROIs (red) for gastrocnemius medialis (C), gastrocnemius lateralis (D), and soleus (E) acquired throughout the experiment for distal (blue) and proximal (orange) slices. Maps are derived from postexercise (60 – 300 s) ^1^H ASL data, as indicated by the red box in the time courses of a representative subject

**Figure 4:**
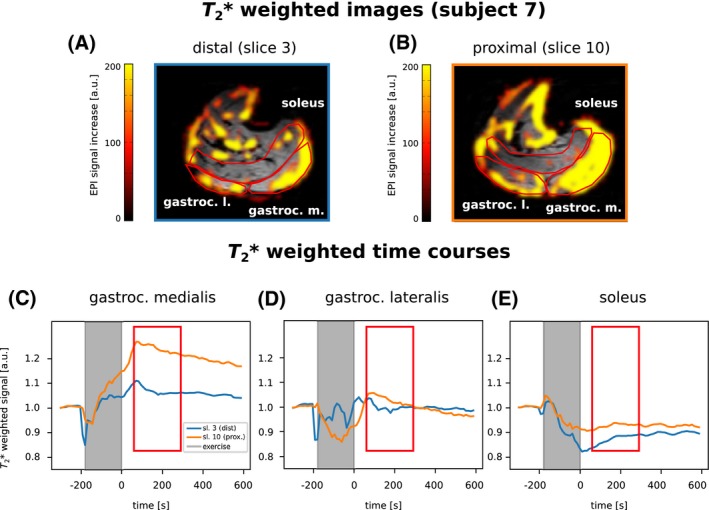
$T_{2}^{*}$‐weighted images at distal (A) and proximal (B) position overlaid with transversal EPI slices of the calf. Corresponding $T_{2}^{*}$‐weighted time courses of three ROIs (red) of gastrocnemius medialis (C), gastrocnemius lateralis (D), and soleus (E). Images were derived from postexercise (60 – 300 s) ^1^H ASL data, as indicated by the red box in the time courses of a representative subject

Simultaneously with ^31^P data acquisition, transversal stacks of ^1^H ASL multi‐slice images were recorded, showing 3D‐resolved perfusion and $T_{2}^{*}$‐weighted signal along the healthy human calf. Postexercise perfusion maps, $T_{2}^{*}$‐weighted images and the corresponding time courses of GM, GL and SOL of a representative subject are shown in Figures 3 and 4 for two slices, at a distal (slice 3) and a proximal (slice 10) position.

**Figure 5:**
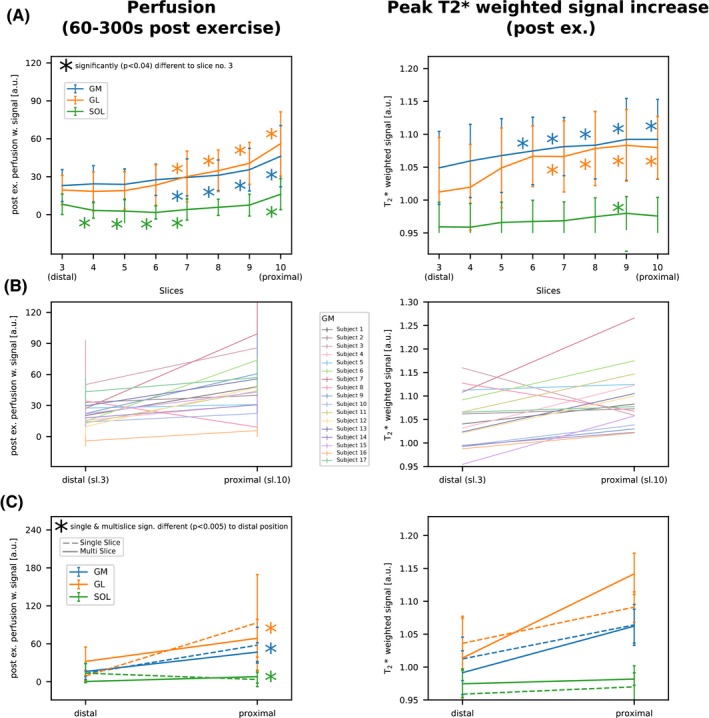
Postexercise (averaged 60‐300 s) perfusion signal and maximum $T_{2}^{*}$‐weighted signal increase of gastrocnemius medialis (blue), gastrocnemius lateralis (orange), and soleus (green) in slices 3 – 10 averaged over all subjects (A). Individual results of GM from all subjects for slices 3 and 10, respectively (B). Comparison of results between multi‐slice (solid line) and single‐slice (dashed line) protocol from four subjects measured additionally on a separate day. Distal and proximal position of the single‐slice acquisition correspond to slice 4 and 10 in the multi‐slice protocol (C)

Averaged postexercise ^1^H MRI results from slices 3 – 10 are presented in Figure 5A for all three muscles over all subjects. Additionally, per‐subject results of GM of slice 3 and 10, are shown in Figure 5B.

Significant differences (*P* < .001) in the parameters derived from ^1^H MRI were found between slice 3 through 10 in GM, GL and SOL, across all subjects: A pairwise comparison between slices in GM, GL and SOL using Nemenyis post hoc tests showed that postexercise perfusion was significantly higher (*P* < .002) in proximal regions (slice 10) than at a distal location (slice 3). GM and GL also showed proximally (slice 10) significantly higher postexercise $T_{2}^{*}$‐weighted peaks (*P* < .005) than distally (slice 3). Results of the multi‐slice protocol were consistent with data acquired using repeated single‐slice ASL measurements in four remeasured subjects (see Bland‐Altman plots in Supporting Information Figure S1). The inter‐method reliability test^36^ showed moderate to strong correlations (*r* = .81, *P* < .001 for postexercise perfusion, and *r* = .67, *P* < .001 for peak $T_{2}^{*}$‐weighted results). These results suggest good reliability of the multi‐slice acquisition protocol. Proximo‐distal differences in perfusion and $T_{2}^{*}$‐weighted signal were found with multi‐slice, as well as with repeated single‐slice ASL MRI, see Figure 5C. Both values were higher proximally than distally in GM and GL. Analysis of test‐retest reliability as a measure of the repeatability of the interleaved ^1^H ASL/^31^P MRS protocol showed strong correlation for postexercise perfusion (*r* = .88, *P* < .001) and $T_{2}^{*}$‐weighted peak (*r* = .83, *P* < .001), see Bland‐Altman plots in Supporting Information Figure S2.

### 3.3 ^31^P MRS versus ^1^H MRI

**Figure 6:**
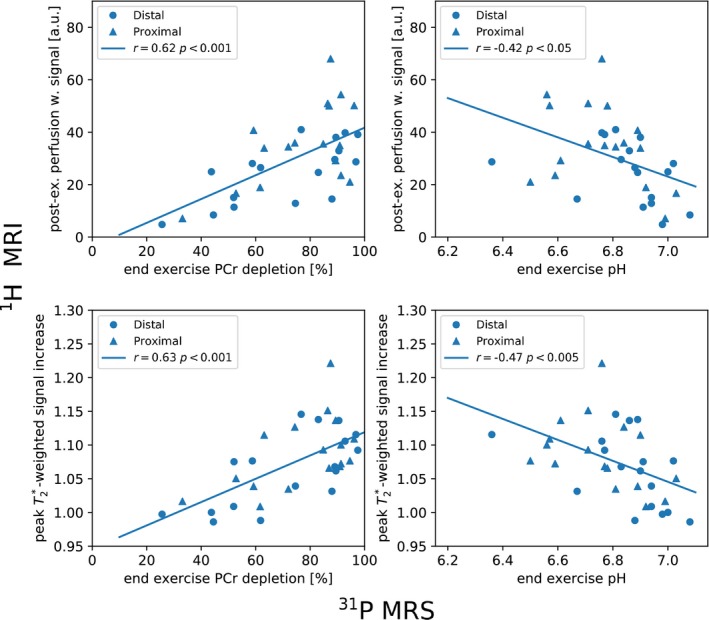
Postexercise (60‐300 s averaged) perfusion signal (top), maximum $T_{2}^{*}$‐weighted signal increase during recovery (bottom) versus end‐exercise PCr depletion (left) and versus end‐exercise pH (right) for distal (circle) and proximal (triangle) position for each subject. Linear regressions were calculated between ^31^P and corresponding ^1^H data in GM over all subjects. For ^1^H results, distal and proximal position represents averaged slices 3‐6 and 7‐10, respectively

Correlations were found between parameters derived from ^31^P and ^1^H data acquired in GM. End‐exercise PCr depletion correlated positively with postexercise perfusion (*r* = .62, *P* < .001) and the maximum $T_{2}^{*}$‐weighted signal increase (*r* = .63, *P* < .001). End‐exercise pH correlated negatively with postexercise perfusion (*r* = −.42, *P* < .05) and maximum $T_{2}^{*}$‐weighted increase (*r* = −.47, *P* < .005), see Figure 6.

## 4 DISCUSSION

The purpose of this study was to present a method that advances investigation of exercising muscle tissue by simultaneously obtaining complementary time‐ and 3D‐resolved multi‐nuclear MR data. The benefit of the approach is its potential to confront information on oxidative phosphorylation with blood perfusion and $T_{2}^{*}$ contrast. To our knowledge, 3D‐resolved dynamic perfusion images, acquired with multi‐slice ^1^H ASL MRI and localized high‐energy metabolite time courses from multi‐voxel ^31^P‐MRS, have never been acquired simultaneously during a single time resolved measurement at ultra‐high field.

Functional and metabolic heterogeneity along a single muscle group has been found recently in a study using ^31^P MRS and ^1^H MRI^25^, ^26^ during multiple successive exercise recovery experiments in tibialis anterior at 3 T. The simultaneous acquisition of complementary data, in combination with the the high specificity of a multi‐slice and multi‐voxel protocol and increased sensitivity of measurements at 7 T, allow for an advanced investigation of intramuscular heterogeneity in functional and metabolic exercise response along human calf muscles.

Gastrocnemius and soleus are the main contributors to force during plantar flexion,^12^, ^14^ but with a straight knee the contribution of gastrocnemius is dominant and its metabolic response to exercise is more pronounced, compared to soleus. For comparing metabolic parameters between distal and proximal parts of one muscle reliably, using localized MRS, the ^31^P VOI sizes should be similar and not too small to maintain sufficient SNR. In the region of maximum calf diameter the cross‐section of GL decreases considerably within a few centimetres towards distal direction, which prevented placement of a sufficiently large ^31^P VOI, hence GM was chosen as target. Despite orienting and aligning the voxels so that only planes selected by refocussing pulses overlapped and avoiding offset, a signal drop on the order of 10 – 15% is inherent to the chosen multi‐voxel approach with symmetric $T_{R}$ similar to $T_{1}$ (cf. Table 1 in^33^).

To allow for a large tagging volume, the slice package was placed in the distal half of the effective coil length. In the majority of subjects decreased SNR was observed in slices 1 and 2 (at the distal end and close to the edge of the coil). This was presumably due to a lower effective $B_{1}$ in this region and, as a consequence, the corresponding perfusion time courses were not reliable. We therefore excluded the two most distal slices from our ASL results analysis. Multiple comparison analysis between all remaining ^1^H slices showed significant differences relative to slice 3 (see Figure 5A). Differences between the most distant slices (3 and 10) were the most evident and hence were used for illustration (see Figure 5B). For the comparison between ^1^H and ^31^P data in Figure 6, results derived from imaging slices 3‐6 and 7‐10 were averaged to match the volumes of the ^31^P voxels accordingly.

Interleaving ^1^H MRI with ^31^P MRS acquisitions induced an increase of ^31^P SNR due to NOE, which did not reach the full theoretically achievable enhancement, in this setting. This SNR gain comes without cost, as a consequence of combining ^1^H ASL with ^31^P MRS, but optimizing NOE by adding further pulses would be detrimental to ^1^H MRI quality and increase energy deposition. Quantifying the NOE enhancement in a brief calibration measurement (as done here) is also required in case of performing metabolite quantification.

Several differences were found between proximal and distal parts of the muscles: Proximally increased perfusion signal and corresponding higher $T_{2}^{*}$‐weighted peaks during recovery correlated with metabolic activity being proximally higher than distally, as evidenced by simultaneously acquired ^31^P data (cf. Figure 6). In the healthy human calf, a higher metabolic activity, presumably due to a stronger recruitment of muscle fibers (reflected in stronger PCr depletion), requires a higher perfusion to satisfy the oxygen demand for ADP rephosphorylation—also if glycolytic contribution during exercise has lead to acidification, which is known to slow down PCr recovery.^37^ To summarize, the results of ^31^P MRS in GM were consistent with findings from ^1^H MRI.

This consistency of complementary information, together with good reliability of the multi‐slice ^1^H ASL protocol (assessed via consecutively acquired single‐slice measurements, cf. Figure 5C and Supporting Information Figure S1), supports the assumption that apparent local differences of functional and metabolic response to exercise are not likely to be caused by a systematic error but indeed reflect a true physiological effect.

The significantly higher end‐exercise PCr depletion and higher postexercise perfusion found proximally are in good agreement with results of Boss et al.,^25^, ^26^ who used an exhaustive exercise paradigm in tibialis anterior. Although submaximal exercise was performed in our study, significant differences of end‐exercise pH and PCr recovery times were found, in contrast to.^25^, ^26^ This could be explained by the fact that the fraction of oxidative Type I muscle fibers is higher in the tibialis anterior which is considered to work in a more oxidative regime compared to the GM.^38^ A finding supporting this is that SOL, a muscle with a high fraction of Type I fibers, does not reach similar acidification as GM, at comparable end‐exercise PCr depletion.^14^ For GM, which contains predominantly glycolytic Type II muscle fibers,^38^ substantial activation is associated with pronounced acidification. It was shown in literature that PCr recovery kinetics is sensitive to exercise intensity^24^ and can be prolonged by acidification.^37^

Although the measured PCr recovery rates were not identical, yet similar rates were obtained for very different levels of muscle perfusion. This suggests that a higher mitochondrial oxidative phosphorylation demand in some territories, which show stronger end‐exercise PCr depletion and stronger acidification, is compensated by an increased blood and oxygen supply to meet the required oxygen consumption. With increased oxygen supply through higher perfusion, one would expect accelerated PCr recovery in an oxidative regime.^39^, ^40^, ^41^ However, stronger acidification proximally (known to slow down PCr recovery) likely dominates over this effect, and we observe slightly but significantly prolonged PCr recovery times.

The fact that a lower perfusion regime was associated with a lesser $T_{2}^{*}$‐weighted response together with a lower metabolic response (less end‐exercise PCr depletion), for similar mitochondrial ATP production, is a strong indicator of data coherence. This consistency was found despite the shortcoming that perfusion signals were only semi‐quantitative due to the limited tagging capability of the relatively short coil available. A lower tissue blood flow might, in principle, be compensated by higher oxygen extraction to maintain oxygen consumption and fuel the mitochondrial oxidative phosphorylation, but the effect of pH on $T_{2}^{*}$‐weighted signal intensity might contribute significantly to the observed signal changes.^42^, ^43^

However, postexercise perfusion and peak $T_{2}^{*}$‐weighted signal increase was found to be heterogeneous along all muscle groups, but it was less pronounced in SOL than in gastrocnemius. A variation in fiber type composition (note that gastrocnemius is a pennated muscle, that is, its fibers do not run from end to end) and/or changes in capillary density along a specific muscle could explain such inhomogeneities, but so far this was only confirmed for rats^44^ and we are not aware of any studies performed in humans. Despite the normalization to maximum voluntary contraction force, inter‐subject variability in end‐exercise PCr depletion was high, which has been already reported by Bendahan et al and Mattei et al.^45^, ^46^

A focus of future studies could be on studying glycolytic muscle at low exercise intensity, potentially over a larger distance (requiring a longer,homogeneous coil if using PASL).

## 5 CONCLUSION

The successful implementation of temporally interleaved multi‐slice ^1^H ASL and multi‐voxel ^31^P MRS at ultra‐high field was applied to simultaneously investigate the metabolic and functional response to plantar flexion exercise along the healthy human GM. Regional metabolic differences along GM, that is, significantly higher PCr depletion and pH drop proximally, correlated with significantly higher perfusion in proximal sections, compared to distal sections. We have successfully shown a feasible way to assess complementary data relevant to physiologic parameters of skeletal muscle at ultra‐high magnetic field, increasing both sensitivity and specificity. This unique approach could provide a more comprehensive picture on muscle metabolism and the interplay between perfusion, oxygen supply and energy consumption. Finally, it could be used to investigate local variations during disease progression in patients suffering from diabetes mellitus, peripheral arterial disease, or muscular diseases such as Duchenne muscular dystrophy.

## Supporting information


**FIGURE S1** Inter‐method reliability of the multi‐slice acquisition scheme verified against single‐slice acquisitions that were repeated at two locations, in four subjects, each. Pearsons correlation coefficients and *p*‐values are given together with Bland‐Altman plots for comparison between the results of postexercise perfusion (A) and the peak $T_{2}^{*}$‐weighted signal (B), obtained with the two acquisition schemes. The 24 data points represent 3 ROIs (placed in GM, GL, and SOL), 2 slice positions, and 4 subjects
**FIGURE S2** Test‐retest reliability as measure of the repeatability of the interleaved ^1^H ASL/^31^P MRS protocol was analyzed in two subjects who were measured twice. Pearsons correlation coefficients and *p*‐values are given together with Bland‐Altman plots for comparison between the results of postexercise perfusion (A) and the peak $T_{2}^{*}$‐weighted signal (B). The 48 data points represent 3 ROIs (placed in GM, GL and SOL) in 8 slices and 2 subjects
**TABLE S1** Analysis of test‐retest reliability of ^31^P MRS. The results of two measurements, which were performed on different days, are given as mean ± SD for each VOI and for the two subjects remeasured
