# Supplementary material for: Interleaved 31P MRS/1H ASL for analysis of metabolic and functional heterogeneity along human lower leg muscles at 7T
Source: Magn Reson Med. 2019 Dec 17;83(6):1909–19. doi: 10.1002/mrm.28088 (PMC7065182; doi:10.1002/mrm.28088)
Supplement: Supplementary file 1 — FIGURE S1 Inter‐method reliability of the multi‐slice acquisition scheme verified against single‐slice acquisitions that were repeated at two locations, in four subjects, each. Pearsons correlation coefficients and p‐values are given together with Bland‐Altman plots for comparison between the results of postexercise perfusion (A) and the peak T2*‐weighted signal (B), obtained with the two acquisition schemes. The 24 data points represent 3 ROIs (placed in GM, GL, and SOL), 2 slice positions, and 4 subjects FIGURE S2 Test‐retest reliability as measure of the repeatability of the interleaved 1H ASL/31P MRS protocol was analyzed in two subjects who were measured twice. Pearsons correlation coefficients and p‐values are given together with Bland‐Altman plots for comparison between the results of postexercise perfusion (A) and the peak T2*‐weighted signal (B). The 48 data points represent 3 ROIs (placed in GM, GL and SOL) in 8 slices and 2 subjects TABLE S1 Analysis of test‐retest reliability of 31P MRS. The results of two measurements, which were performed on different days, are given as mean ± SD for each VOI and for the two subjects remeasured [file MRM-83-1909-s001.pdf]

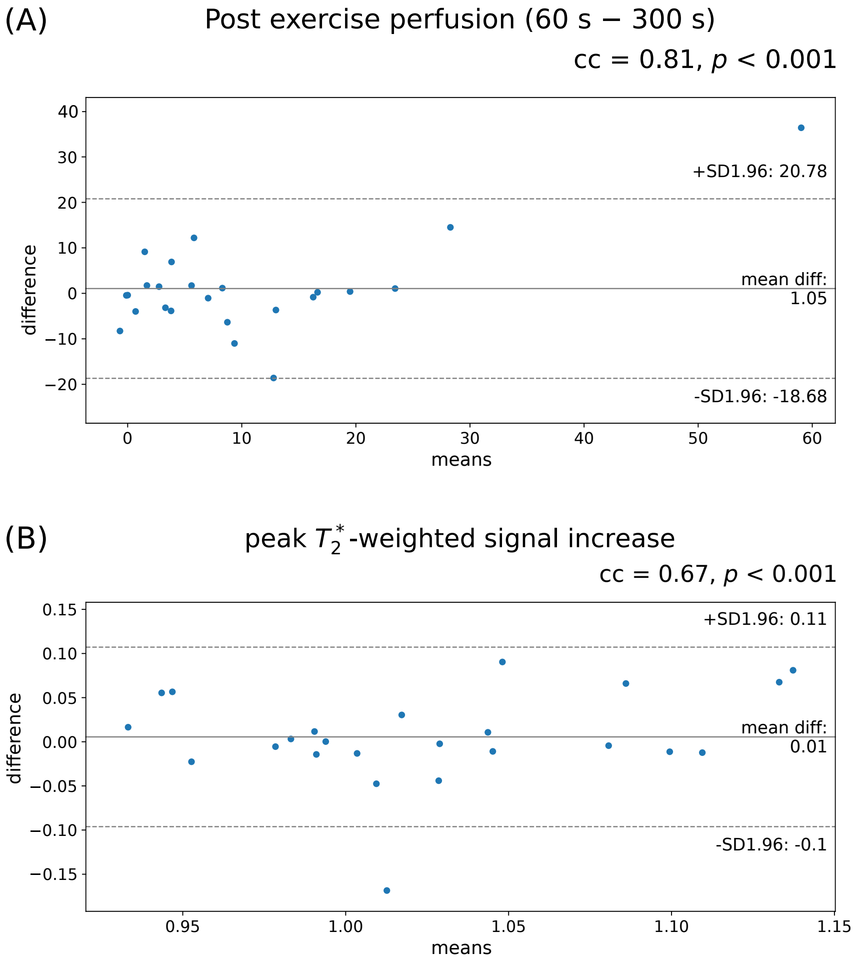

Figure S1: Inter-method reliability of the multi-slice acquisition scheme verified aganist single-slice acquisitions that were repeated at two locations, in four subjects, each. Pearsons correlation coefficients and p-values are given together with Bland-Altman plots for comparison between the results of post exercise perfusion (A) and the peak T_2^*-weighted signal (B), obtained with the two acquisition schemes. The 24 data points represent 3 ROIs (placed in GM, GL and SOL), 2 slice positions and 4 subjects.

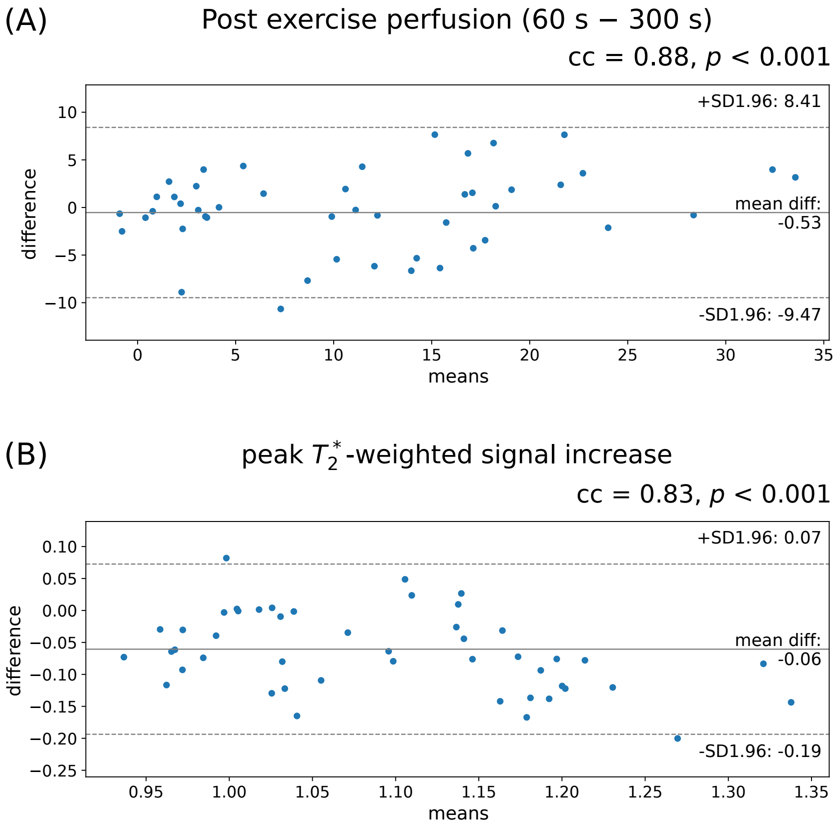

Figure S2: Test-retest reliability as measure of the repeatability of the interleaved ^{1}H ASL / ^{31}P MRS protocol was analysed in two subjects who where measured twice. Pearsons correlation coefficients and p-values are given together with Bland-Altman plots for comparison between the results of post exercise perfusion (A) and the peak T_2^*-weighted signal (B). The 48 data points represent 3 ROIs (placed in GM, GL and SOL) in 8 slices and 2 subjects.

Table S1: Analysis of test-retest reliability of ^{31}P MRS. The results of two measurements, which were performed on different days, are given as mean ± SD for each VOI and for the two subjects remeasured.

	Subject 6		Subject 7	
^{31}P MRS	GM_{distal}	$GM_{proximal}$	GM_{distal}	$GM_{proximal}$
PCr depletion [%]	88 ± 4	89 ± 3	83 ± 5	91 ± 4
pH_{ee}	6.79 ± 0.001	6.73 ± 0.02	6.8 ± 0.05	6.68 ± 0.08
τ_{PCr} [s]	39 ± 6	45 ± 0	34 ± 11	37 ± 6
